# Aesthetic preference is related to organized complexity

**DOI:** 10.1371/journal.pone.0235257

**Published:** 2020-06-26

**Authors:** Alexandros A. Lavdas, Uta Schirpke

**Affiliations:** 1 Institute for Biomedicine, Eurac Research, Affiliated Institute of the University of Lübeck, Bolzano, Italy; 2 Institute for Alpine Environment, Eurac Research, Bozen/Bolzano, Italy; 3 Department of Ecology, University of Innsbruck, Innsbruck, Austria; Northwestern University, UNITED STATES

## Abstract

There is extensive evidence today linking exposure to natural environments to favorable changes in mental and even physical health. There is also a growing body of work indicating that there are specific geometric properties of natural scenes that mediate these effects, and that these properties can also be found in artificial structures like buildings, especially those designed before the emergence of modernism. These geometries are also associated with aesthetic preference–we seem to like what is good for us. Here, using a questionnaire-based survey, we have tried to elucidate some of the parameters that play a role in formulating a preference for one form over the other. The images used were nature scenes from the Alpine landscape with various manipulations to alter their complexity, or with additions of computer graphics or various buildings. In all cases, the presence of a natural scaling hierarchy and of either fractal graphics or of ornate, non-local pre-modern buildings was always preferable to the alternative. We discuss these findings under the light of recent evidence in the field and conclude that they support the idea of the existence of a preference of our perceptive system for certain types of visual organization.

## Introduction

Starting from the classic work of Hubel and Wiesel [1, 2] the effect of visual input on the visual system has been well documented, and the physiological influence of visual and other environmental enrichment on the brain at the molecular, anatomical and functional level has been studied extensively (for reviews, see [3–5]). In recent years, many researchers have turned their attention to the potential physiological impact of the visual organization of our surroundings, either natural or artificial [6–9]. “Naturalness”, a term used to identify “structural” morphological properties of our environment, has attracted particular attention; for review, see: [10]. Natural surroundings have been shown to exert positive effects (sometimes by simple viewing of images) in mood [11], reducing stress [12], improving concentration and working memory [13–17], self-perceived health [18] and even, amazingly, self-esteem [11, 19]. Other intriguing findings include the association of exposure to natural forms to a reduction of criminal behavior [20, 21] and the, now famous, finding of improved recovery from surgery [22].

These findings support the “Biophilia” hypothesis, which suggests that humans have an innate affinity for other living organisms and the natural environment in general [23, 24], and they indicate that exposure to such environment can affect mental and physical health. Consequently, investigating the “structural” visual features that, presumably, mediate these effects, is a task of both basic scientific and practical importance. These features are not only to be found in nature, but also in artificial environments, either as a result of a conscious effort to incorporate them in a “biophilic” type of design or–perhaps more interestingly—in pre-modern architecture. In the latter, such characteristics are embedded as an integral part of the design or, in the simplest of cases, they emerge from the materials and the construction methods used. It has been proposed that exposure to this type of visual organization even in artificial environments may have the same positive effects [9, 25–27]. Closing the circle, the existence of this structural order in traditional architecture may in fact be a reflection of structural and functional patterns of our own nervous system [28]. Evolving exposed to (and as part of) the geometry of nature, seems to have had a profound impact on the way that we connect to natural forms. Pioneering work of Alexander [29] and Salingaros [30–33] has identified a number of parameters that codify this “connectedness” between the viewer and the environment. One important factor is the presence of fractal features. The term fractal, introduced by Mandelbrot [34], denotes a structure that exhibits self-similarity on different levels, from the largest to the smallest. Perfect fractals are purely mathematical constructs, but statistically self-similar fractals, where the repetitions are not exact, are everywhere in nature, from coastlines to galaxies and from tree branches to pulmonary airways and blood vessels [35–37]. It has been shown that exposure to certain fractal visual patterns in nature, architecture or visual arts has significant physiological effects [24, 27, 38, 39]. However, there is more to these qualities of the natural or “biophilic” environment than their fractal properties. Our processing “… system is acutely tuned to the visual complexity of the natural environment, specifically to respond positively to the highest levels of organized complexity. Fractals are an important component of this effect, but by no means represent the full gamut of connective qualities” [40]. This “organized complexity” is also defined by a hierarchy of scales, the presence of local contrasts, as well as overall coherence etc.

Studies in recent years have contributed to our understanding of our interaction with specific visual features of the environment by documenting reactions to images, analyzing those images for a number of features, and analyzing the two sets of data to establish consistent correlations [41, 42]. Here, we have attempted to follow the opposite course, i.e. to create images that contain certain features, and then record the reactions to those features using a questionnaire-based ratings assay.

## Materials and methods

### Conceptual basis

The main goal was to tease out the different parameters of complexity and test for them separately. To this end, we used i) insertion of simple, computer-generated structures, either non-fractal or fractal of different fractal dimensions, ii) changes in scaling hierarchy in natural elements in processed images, iii) addition of forms, either natural or built, simple or highly complex and at the same time not familiar for the particular environment–thus also teasing out aesthetical preference from familiarity [43, 44].

The Alpine landscape was used as a common visual substrate for all these forms and manipulations. This allowed us to study the effects of changing only specific parameters of the image each time, as opposed to comparing between different natural or urban scenes. Our working hypothesis was that a difference in the perception of beauty would be the main outcome of these manipulations. To more accurately circumscribe the reception of these manipulations by the participants in the survey, we also examined changes in whether an image was deemed as interesting, “making sense” or familiar. We expected image versions that were deemed more beautiful to be also seen as more interesting, because of the increased amount of information they presented for the viewer to explore [43]. The question about whether an image is “making sense” was used as a way to assess what has been described as coherence in environmental psychology [44–46]. An orderly setting that is organized into a "few distinct regions or areas" that have "some repeating themes and unifying textures … and a limited number of contrasting textures" defines coherence [46]; coherence allows a scene to "hang together" [45]. Familiarity was assessed to exclude the possibility that a scene was preferred not because of its properties, but because it was reminiscent of other, familiar, scenes.

### Image manipulations

**Fig 1:**
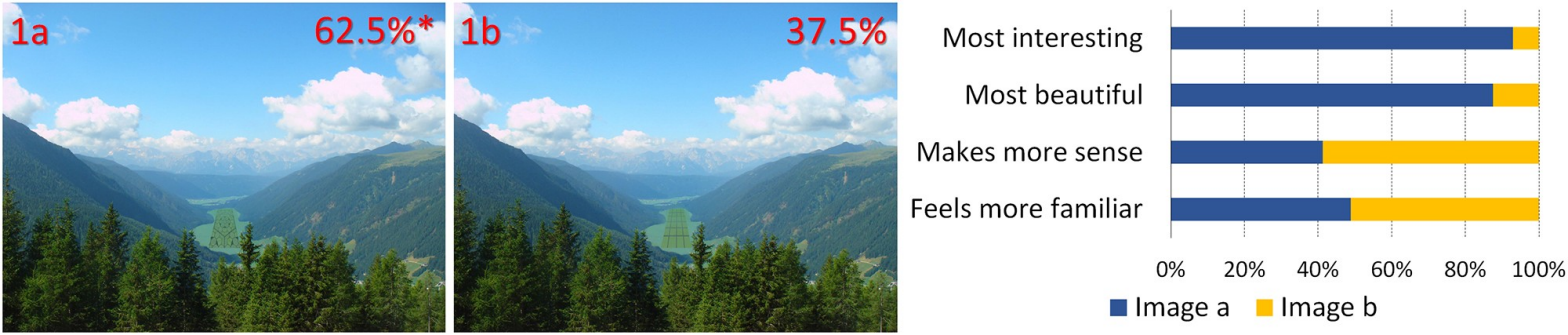
Linear and fractal additions to a valley. Percentages represent the preferences and the asterisk indicates a statistically significant difference in overall preference (image adapted from Schirpke et al, 2013, https://www.mdpi.com/2071-1050/5/3/1080/htm, CC BY 3.0 license).

**Fig 2:**
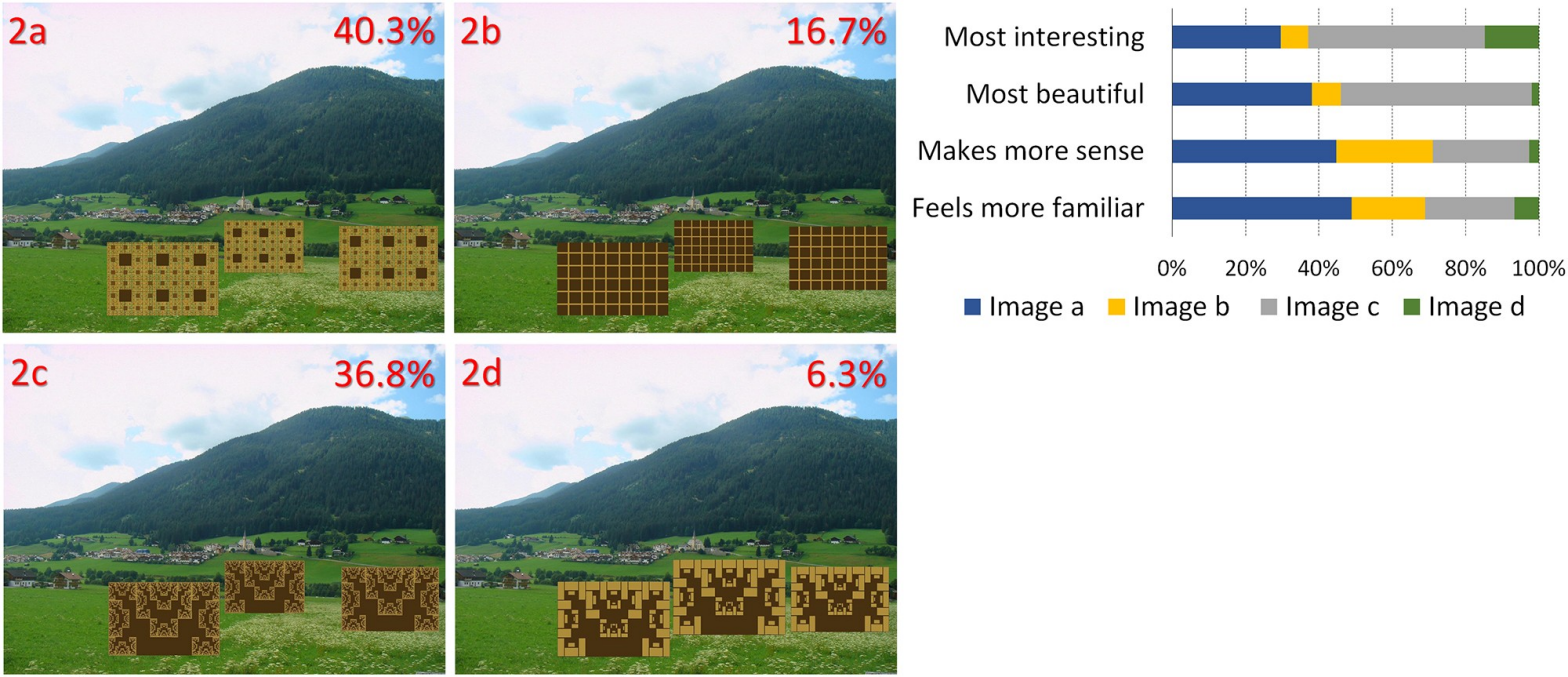
Computer graphics added to a village view. Percentages represent the preferences and the asterisk indicates a statistically significant difference in overall preference from all other images (image adapted from Schirpke et al, 2013, https://www.mdpi.com/2071-1050/5/3/1080/htm, CC BY 3.0 license; graphs in 2c, 2d reprinted from http://barlior.com/fractals/fractals.htm under a CC BY license, with permission from Lior P Bar, original copyright 2003).

**Fig 3:**
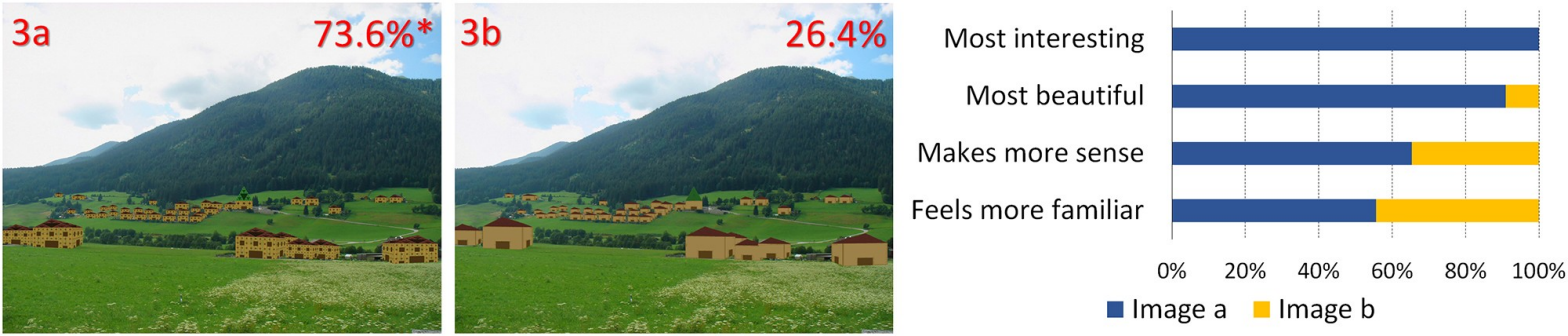
Computer graphics added to a village view. Percentages represent the preferences and the asterisk indicates a statistically significant difference in overall preference (image adapted from Schirpke et al, 2013, https://www.mdpi.com/2071-1050/5/3/1080/htm, CC BY 3.0 license).

**Fig 4:**
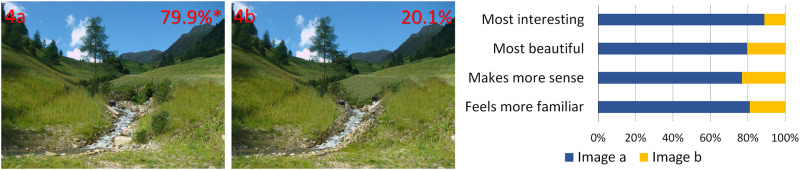
Changes in complexity and scaling hierarchy of real objects. Percentages represent the preferences and the asterisk indicates a statistically significant difference in overall preference (image by AA Lavdas).

**Fig 5:**
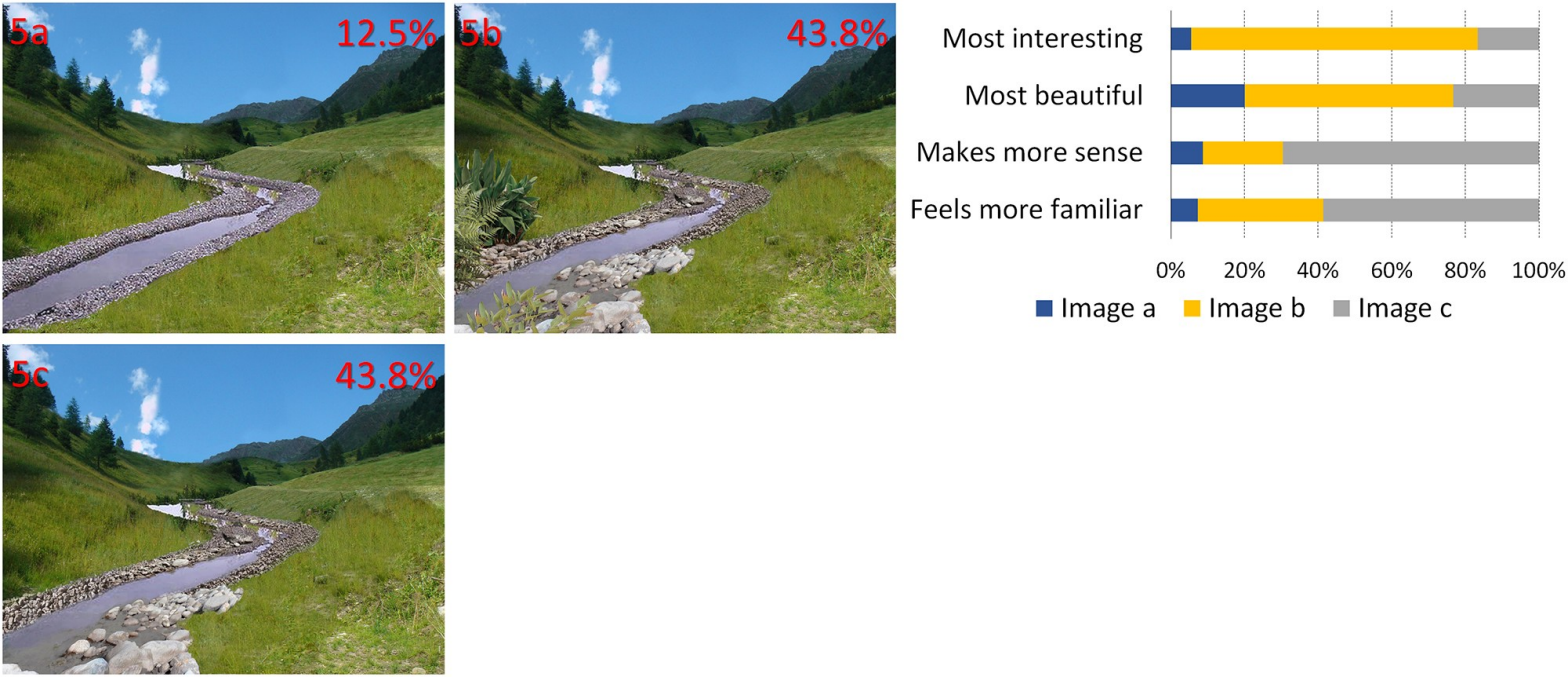
Changes in complexity and scaling hierarchy of real objects. Percentages represent the preferences (image by AA Lavdas).

**Fig 6:**
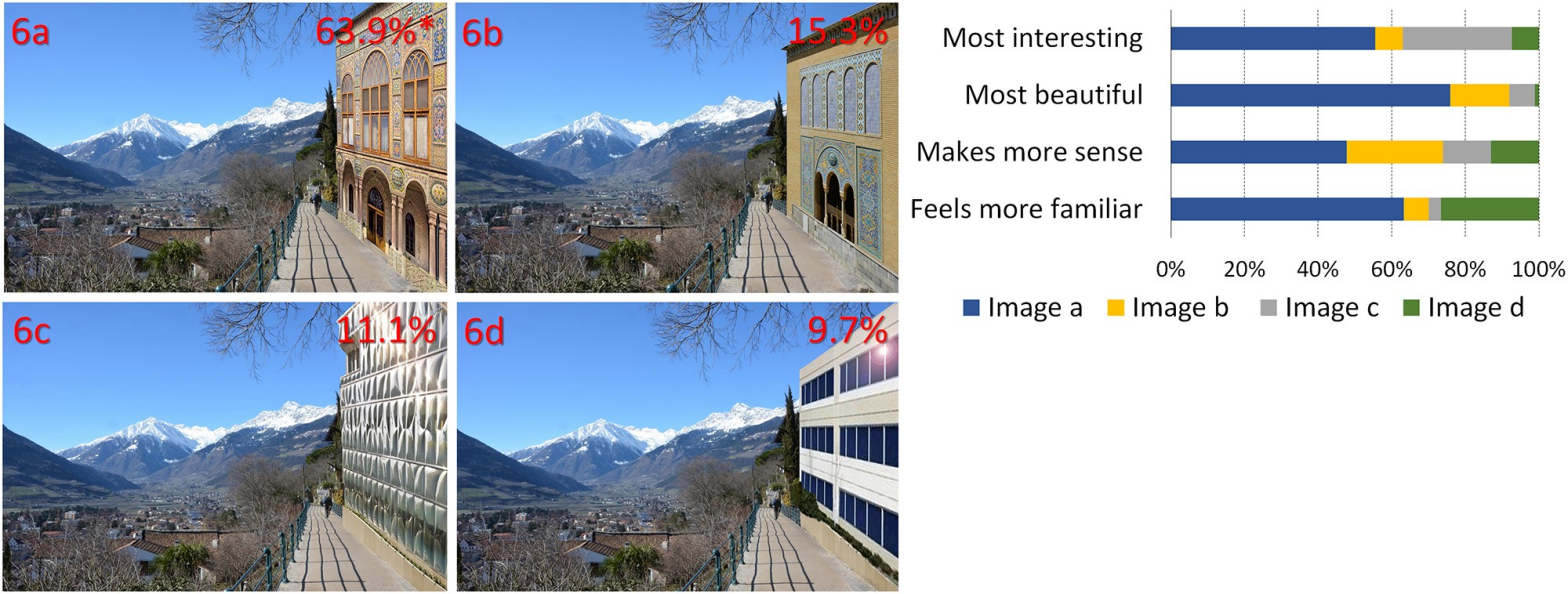
Addition of modern and non-local traditional buildings to the Alpine landscape. Percentages represent the preferences and the asterisk indicates a statistically significant difference in overall preference from all other images (images by AA Lavdas; building image in 6c reprinted from https://i1.wp.com/www10.aeccafe.com/blogs/arch-showcase/files/2018/06/9800Wilshire_Photo-%C2%BCBruceDamonte_05.jpg?ssl=1 under a CC BY license, with permission from Bruce Damonte, original copyright 2014).

**Fig 7:**
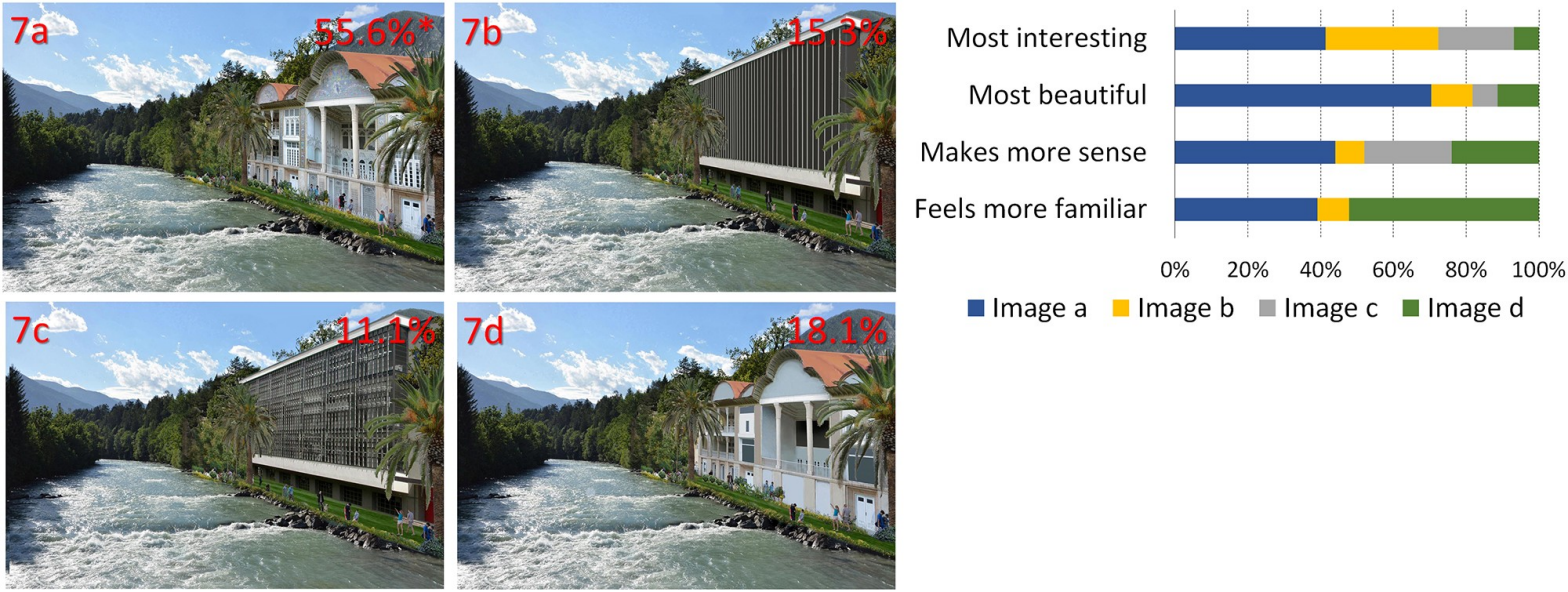
Addition of modern and non-local traditional buildings to the Alpine landscape, in their original and processed form. Percentages represent the preferences and the asterisk indicates a statistically significant difference in overall preference from all other images (images by AA Lavdas).

A number of images were used, all from the region of Tyrol in Austria and Italy, to collect people’s preferences using an image-based questionnaire (Figs 1–7). The images were subjected to various manipulations, using Adobe Photoshop image processing software. The manipulations were related to:

1. Fractal and non-fractal geometric forms: adding computer graphics of different geometries,
2. Scaling order: manipulation of scaling order of the scene by manipulation and insertion of natural objects and
3. Building style and complexity: insertion of buildings of different styles and manipulation of their complexity (Table 1).

**Table 1:** Outline of image manipulations.

Figure	Manipulation group	Manipulation
**1**	**A**	Insertion of computer-generated graphics of different complexity embedded in the scene
**2**	**A**	Insertion of computer-generated graphics of different complexity standing out as artificial
**3**	**A**	Insertion of computer-generated graphics of different complexity standing out as artificial
**4**	**B**	Manipulation of scaling order of the scene by manipulation of natural objects
**5**	**B**	Manipulation of scaling order of the scene by manipulation and insertion of natural objects
**6**	**C**	Insertion of buildings of different styles with different levels of complexity
**7**	**C**	Insertion of buildings of different styles with different levels of complexity and manipulation of their complexity

More specifically (see also Table 1):

- In Fig 1, the image of the valley between the mountains has been altered in both cases through the insertion of patterns that have been fitted to the image perspective, to seem like they are actually in the valley. Inserts have been color-matched. In 1a, there is a fractal pattern, whereas in 1b the pattern is rectilinear.
- In Fig 2, artificial, color-matched, image elements were added to the image of a Tyrolean village. In 2a, it was part of a Sierpinski carpet, (a perfect fractal [47]), in 2b a simple rectilinear pattern. In the next two images, we added a more advanced (2c) and a less advanced (2d) fractal iteration of the letter “E" at a 90 degree angle. The inserted items are artificial-looking, so that they are not expected to be perceived as real objects.
- For Fig 3, we used the same image as Fig 2, but all the buildings of the village were replaced by artificial patterns, either half a Sierpinski carpet (3a) or a box of the same size in which all elements of the Sierpinski carpet had been removed, except the central box (3b). A roof-like triangular shape was added to all the boxes; in the first group, it was a Sierpinski triangle (also a perfect fractal, [48]), in the second, it was a flat-colored triangle.
- Fig 4 depicts a stream and its surroundings, presented either unaltered (4a) or modified (4b). The modification consisted in replacing most of the rocks, which had various sizes, with multiple copies of a few rocks, and also reducing the variety in the vegetation next to the stream.
- The image used in Fig 4 was heavily modified for Fig 5. In all three versions, the tree was removed, and the stream was replaced by an artificial stream with identical rocks on its sides. In 5c, a set of rocks of various sizes was added, and in 5b, in addition, there is a set of plants that were not native to the environment.
- In Fig 6, four versions of one image were created by the addition of a building. In 6a and 6b, the buildings were part of the Palace complex of Golestan in Tehran, Iran. In 6c and 6d, the buildings were contemporary office buildings.
- In Fig 7, four versions of the image were created by the addition of a building. In 7a and 7c, the building was the mansion in Eram Gardens in Shiraz, Iran. In 7c, the building was simplified by replacing frescoes with flat color, erasing carving, replacing elaborate railings with flat surfaces etc. The building in 7b and 7d was the Bauhaus building in Dessau, Germany. Transparent windows were replaced with flat dark gray/black areas in 7b.

The graphics used in Figs 1–3 were subjected to fractal analysis for determining their fractal dimension D, a mathematical measure of the fractals’ internal scaling (Mandelbrot, 1977) ranging from 1 to 2. To this end, we used *Image J* software (https://imagej.nih.gov/ij/index.html), a public domain Java-based image-processing and analysis program developed at the *National Institutes of Health* and the *Laboratory for Optical and Computational Instrumentation*. The box-counting method, a widely used method for fractal analysis, was employed. The purpose of this analysis here was to know exactly what was added in the figures, after taking into account resolution limits, potential artifacts etc. The analysis was then performed on cropped portions of the actual images used in the survey at the resolution used. This analysis was carried out only for Figs 1–3, where fractals of various complexity as well as non-fractal graphics were added, to establish their fractal dimension in the final figures (S1 Fig). For the other images, the parameters involved are more diverse and calculation of fractal values is more challenging. Also, given the limitations of the box-counting method [49], these numbers should be seen as a means of comparison between the different elements, rather than as absolute values.

### Online survey and analysis

The seven image sets were arranged in an online questionnaire. After a short introduction, each picture set was shown on an individual page, asking the participants to indicate which image they preferred. Additionally, we asked what made them choose this image by offering the following four options: “Most beautiful”, “Most interesting”, “Makes more sense” and “Feels more familiar”. Respondents could indicate more than one option. These questions were chosen to tease out aesthetic preference from familiarity, and to further differentiate between aesthetic and intellectual appeal, if such a distinction could be made. Finally, we asked the participants to provide socio-demographic information (gender, age, and place of living). The questionnaire was made available between February and June 2019 in three languages (English, German and Italian). The links to the questionnaires were distributed via social media channels and by sending invitations via email.

All answers were registered in a database. We analyzed all valid answers separately for each image set by calculating preference scores and by counting how often participants rated one of the four options for each image. To identify significant differences among the image preferences and the four options, we carried out paired t-tests using SPSS software package (version 25, IBM).

### Ethics statement

The topic is not ethically sensitive and was carried out in accordance with national and institutional legal and ethical requirements. Data were collected completely anonymously (i.e. no possibility to reidentify whatsoever) and therefore this work falls outside the scope of GDPR 2016.

The project follows institutional guidelines and was discussed with the internal ethics reference person who indicated that there was no need for ethical approval when surveys are not directly health related. In Italy, there is no legal requirement for ethical approval of such a survey where no sensitive issues are explored and no privacy is involved, and there are no IRB mechanisms in place for this type of work. Sensitive data or research involving human subject undergo ethical approval through ethical research committees based in hospitals that do not assess this type of projects.

Also, ethical concerns were assessed internally: participation was on a voluntary basis and all participants were informed that the survey was anonymous, that all data would be only used for research and evaluated anonymously. To secure privacy, all data was collected via a web survey with no collection of identifiers/codes and analyzed therefore anonymously. In particular, aggregation was already built into the questionnaire (age groups, world regions, etc.) and no IP addresses were collected.

## Results

From the online survey, we received 144 valid responses. Female respondents prevailed over male respondents (for details, see S1 Table in S1 File). Most respondents were middle-aged and lived in large cities, but participants of all age groups and different living places were present. A proportion of 81.9% of the participants stated that they lived in Europe, 11.8% in other parts of the world and 6.3% gave no response. The obtained preference scores from the responses showed in most cases a clear preference for one image (see also Figs 1–7). Statistically significant preferences were obtained for images 1a, 3a, 4a, 6a and 7a. For Fig 2, image 2a was selected most often, followed by 2c, both differing significantly from images 2b and 2d. In Fig 5, the more natural looking images 5b and 5c had equal preferences, differing significantly from 5a. A detailed list of the responses can be found in Table 2, and the results of the paired t-test in S2-S4 Tables, in S1 File.

**Table 2:** Image preferences indicated by the respondents (n = 144). Reason for selecting this image expressed through four options (“Most beautiful”, “Most interesting”, “Makes more sense”, “Feels more familiar”), multiple selections were allowed. All values represent number of mentions.

Figure	Image	Preference	Option			
			Feels more familiar	Makes more sense	Most beautiful	Most interesting
1	1a	90	23	19	28	26
	1b	54	24	27	4	2
2	2a	58	22	17	19	8
	2b	24	9	10	4	2
	2c	53	11	10	26	13
	2d	9	3	1	1	4
3	3a	106	30	32	40	22
	3b	38	24	17	4	0
4	4a	115	30	30	55	16
	4b	29	7	9	14	2
5	5a	18	3	4	12	1
	5b	63	14	10	34	14
	5c	63	24	32	14	3
6	6a	92	19	11	66	15
	6b	22	2	6	14	2
	6c	16	1	3	6	8
	6d	14	8	3	1	2
7	7a	80	9	11	62	12
	7b	22	2	2	10	9
	7c	16	0	6	6	6
	7d	26	12	6	10	2

Among the four options what made them choose the image, respondents indicated most often “most beautiful” (37.3%), followed by “Feels more familiar” (24.0%) and “Makes more sense” (23.5%), whereas “Most interesting” was selected least often (15.2%); for details, see Table 2. “Most beautiful” was also significantly higher in Figs 4, 6 and 7, while “Most interesting” differed significantly from the other options for Figs 3, 4 and 5 (S3 Table in S1 File). For Figs 1 and 2, no significant differences among all four options were found.

Although not always statistically significant, some major differences can be found for the individual figures.

**A) Different fractal and non-fractal geometric forms**
  In Figs 1–3, the independent variable was the fractal dimension of the inserted forms. Image 1a was voted more beautiful and more interesting, whereas 1b it was only marginally deemed as more familiar (S4 Table in S1 File). For the second image set, the overall preference was divided between 2a and 2c, with no statistical difference between them. They were both significantly preferable than the other two, with 2a deemed as somewhat more “familiar” and 2c somewhat more “beautiful”. Fig 3 were considered as “more interesting” and “more beautiful”, but only marginally as “making more sense”, or being “more familiar”.
**B) Modified scaling order of the scene**
  In Figs 4 and 5, the independent variable was scaling (4 and 5) and complexity, increased by the presence of unfamiliar vegetation (5). In Fig 4, all parameters, including familiarity, were favored in the unretouched image 4a over image 4b. In Fig 5, image 5c “makes more sense” and “feels more familiar”, but 5b was both “more beautiful” and “more interesting” by a large margin.
**C) Different building styles and complexity**
  In Figs 6 and 7, all the variables that define “organized complexity” differed between the different versions, by insertion of different building types, either in their original form or after processing. In Fig 6, image 6a was deemed significantly as “more beautiful”, while 6d was seen as less “interesting” but “more familiar” of all, and 6c as less “familiar”. Similarly, image 7a was rated as “most beautiful” as well as higher in the options “most interesting” and “makes more sense”, whereas 7d was the most familiar image of all.

## Discussion

Numerous studies in the past several years have focused on morphological properties of our natural and artificial environment, by documenting reactions to images and pursuing to identify the features responsible for those reactions [9, 10, 41, 42]. In this study, we created sets of images that differed in certain, pre-decided morphological features, and then recorded the reception of those images using a questionnaire. The goal of this complementary approach was to allow us to address the effect of these specific features in a controlled manner.

There is one constant pattern in the answers to all questions: both the overall preference and the rating for beauty always favor the version of the photograph with the highest degree of organized complexity. This is not necessarily accompanied by an increase in the familiarity ranking. Qualitatively, the distribution between images of the score for interest resembles that of the score for beauty, while the score for familiarity resembles that for “making more sense”. In the following, we discuss our results in relation to fractals, scaling order and complexity.

**A) Fractal and non-fractal geometric forms**
  In Figs 1–3, there was always a strong preference for the fractal pattern. In Fig 1, if familiarity were the decisive factor, one might expect that 1b would be preferable, as long lines, converging because of perspective, are reminiscent of familiar views of airport runways and highways. However, 1a was voted as both more beautiful and more interesting.
  In Fig 2, the modified Sierpinski carpet pattern could be seen as somewhat reminiscent of a house with windows (but with no door visible–perhaps like a rear view of a house) and this may explain part of its popularity among the respondents. However, looking at the results for all forms, they suggest that this parameter is not the only one–perhaps not even the strongest one–to influence choices: 2c does not resemble any familiar pattern found in buildings, and yet has a similar score. In fact, it was considered the most beautiful and interesting of all versions. It was even considered more familiar than 2b, although 2b does look like a modern building with a curtain-wall exterior. Interestingly, 2d, which is a low-iteration version of the same fractal used in 2c, was the least favorite across the board. This finding directly links the degree of organized complexity with the perceived degree of aesthetic and intellectual attractiveness of a form. In this simplest of cases, in fact, it links it to the fractal dimension of the shape, with higher values being more attractive (see S1 Fig).
  In Fig 3, the overall shape, including the central box, could be seen as reminiscent of a house in both versions, but the internal structure differed. 3a was considered both “more interesting” and “more beautiful”, although only marginally “making more sense”, or being “more familiar”. One might argue that the lack of smaller squares–which could be perceived as windows–is the main source of 3b’s unpopularity. However, the high scores of the more building-like image 2b argue against this and in favor of a choice based on the degree of organized complexity–and, more specifically in these examples, fractal geometry. In the first three images, there was a consistent preference for elements with a higher fractal dimension (D), between 1.64–1.85 (S1 Fig). As already discussed, these values are not to be taken as absolute. However, we can still remark that their high range is in keeping with the findings of Forsythe and colleague [50] for preferences in art, with several examples of very popular great paintings listed as ranging between 1.71 and 1.89. These are much higher than the “optimal” value of around 1.4, discussed by Taylor [27] and Salingaros [25] as stress-reducing. The explanation for this apparent discrepancy lies in more than one reasons, and examining them is beyond the scope of this discussion. It is important, however, to remember that the images we tested did not entirely consist of such patterns; these elements were inserted in the images, and their presence modified the images’ appeal. The fractal dimension of the image as a whole was only slightly modulated (not shown). Immersing participants in virtual reality setups [51, 52], where all parameters of the visual environment can be controlled and their contribution to it can be altered in a defined manner, would be a useful way forward with this type of experiments.
**B) Scaling order**
  In Figs 4 and 5, where the differences consisted of manipulations of real objects, the images fared worse when scaling had been artificially altered to result in narrower ranges of shapes and sizes (4b, 5a). The comparison between 5b and 5c is especially interesting: they both have rocks of various sizes added, so as to simulate a more natural rock size distribution, but 5b also has some tropical vegetation added, which is clearly out of place in the Alpine surroundings. Accordingly, 5c “made more sense” and “felt more familiar”, but 5b was both “more beautiful” and “more interesting”. This, like in previous cases, illustrates the dichotomy between what is intellectually and aesthetically stimulating and what is expected or familiar.
  The aesthetical preference for scenes that display a certain type of complexity in our results, is in keeping with previous work both related to the built environment (see above) and also to the natural environment [53]. For example, naturalness positively influences people’s perceptions of landscape beauty [45, 54], while loss of scenic beauty comes as a result of urban sprawl [55] and an increase in monotone landscapes, such as in agricultural monocultures or reforestation of abandoned mountain grassland [56]. Accordingly, several studies have found that increased perceived scenic beauty values are positively correlated with landscape diversity [57–59]. There are also nonlinear relationships between shape complexity and preferences, i.e. medium levels of shape complexity is preferred over low and high shape complexity [60].
  On the other, most reductionist, end of the spectrum, a questionnaire-based study has also reported findings compatible with our own. When presented with series of various-sized modelling plaster chunks against a dark background, the degree of conformity to Salingaros’ scaling hierarchy law [30] was always proportional to the preference rating: big “scale jumps” were consistently judged as resulting in “uglier” images, while arrangements conforming to a scaling hierarchy were consistently found to be “prettier” [61].
**C) Building style and complexity**
  In Figs 6 and 7, the manipulations, once again, involved real objects, but this time it was buildings, either in their original state or after manipulations, that decreased their complexity. Persian traditional buildings were voted as overall preferable and more beautiful than modern ones, when transplanted in the Tyrolean environment. Traditional Tyrolean buildings were purposely avoided, to preclude familiarity. Traditional Persian buildings are highly ornate and, at the same time, not associated with the Alpine region. Of particular interest is the voting for the two “simplified” versions, 7b and 7d. The original Bauhaus building almost always fares worse than the “simplified” version. This does not contradict our working hypothesis–in fact, it strengthens it: the visual information provided through the glass windows is random, and is perceived as “noise” rather than as conveying a meaningful structure. The simplified version has, at least, an order, albeit a rudimentary one. It may be that the contrast of this order with the lush surroundings (including a palm tree transplanted here from Eram Gardens) makes for an interesting sight–competing with 7a in this respect. The “familiarity” of 7d may be related to the fact that traditional buildings local to the environment are less ornate than their Iranian counterparts, and hence this simplified version seems more familiar. Recent eye-tracking work [62–64] investigated the exploratory movements of volunteers’ gaze when first confronted with different types of images, with some very interesting results, relevant to our findings. It was shown that people look for details, contrasts and, most importantly, structures that can be readily identified and understood. Pre-modern structures with more complexity in the form of decoration etc., are explored in their details, and important features like the entrance are immediately identified. Contemporary buildings are explored in passing, with no clear fixation points–often, incidental features of other structures in the scene attract more attention than the building itself.
  There is an interesting point to be made here: the brain interprets visual information through analyzing statistical patterns of spatial frequencies present in the scene (for review, see [65]), and, intriguingly, the processing of recursive (fractal) forms seems to recruit different resources from the processing of non-fractal stimuli [66]. Whether this applies only to mathematical fractals, as those used by Martins and colleagues [66], and also by us in the first 3 figures, or also to statistical fractals, as those found everywhere in nature [35–37] and also in pre-modern architecture [39], remains to be assessed.

### Limitations

In this type of studies, the choice of images represents an inherent limitation and a potential source of bias, as they are chosen with a certain classification in mind. While this is always a legitimate concern to some degree, we believe that the way this study was structured minimizes its relevance. In contrast to studies concerned with the “natural” or “biophilic” features of the used images, where a choice bias is considered inevitable [41], in our case, the choice was not based on assumptions about the nature of the used images, but on objectively verifiable features manipulated by us. These are the fractal nature (and dimension) of added graphics, the scale hierarchy of natural items and the modern or pre-modern style of the building. This is certainly not discussed here as a criticism for those studies, which have a different objective and thus follow a different experimental outline, but as further clarification for what we have pursued to do. Finally, as 81.9% of the participants stated that they lived in Europe, we cannot claim that the sample is representative on a global level.

### Conclusions and future perspectives

The conclusions of this study, coming from the answers to all questions, can be summarized as follows:

- When artificial forms are inserted in a natural scene, the degree of overall and aesthetical preference correlates with the fractal dimension of the form: forms that are more complex are perceived not only as more beautiful, but also as more interesting.
- Disruption of natural scaling in nature scenes results in scenes that are rated worse.
- Enhancement of natural-scaled complexity through the addition of natural elements enhances the perceived beauty of the scene. This is true even if these elements are foreign to the particular landscape, and are recognized as such (the scene deemed as less familiar and as “making less sense”).
- Addition of buildings with a high degree of organized complexity results in images that are always preferable and deemed as more beautiful than images where the added buildings do not display this feature. This is independent of the familiarity of the particular building style.

These findings support the idea of the existence of a preference (or “tuning”) of our perceptive system to certain types of visual organization. At the same time, in conjunction with previous findings, they underline the urgency for a new approach to the management of our natural and built environment. This is, in essence, a study in empirical aesthetics. However, the approach of isolating individual parameters of complexity and examining their effect on the viewer is used as a first step, with the idea that it should be expanded with studies focusing on physiological readouts, and it is our intention to pursue this investigation in this direction.

## Supporting information

S1 Fig Calculation of fractal dimension of inserted artificial elements, as they appeared in the images used for the survey. (TIF)

S1 File Demographic characteristics of the respondents and statistical analyses of the results. (PDF)

## References

[1] Wiesel TN, Hubel DH. Effects of Visual Deprivation on Morphology and Physiology of Cells in the Cats Lateral Geniculate Body. J Neurophysiol. 1963;26:978–93. 10.1152/jn.1963.26.6.978 14084170

[2] Wiesel TN, Hubel DH. Single-Cell Responses in Striate Cortex of Kittens Deprived of Vision in One Eye. J Neurophysiol. 1963;26:1003–17. 10.1152/jn.1963.26.6.1003 14084161

[3] van Praag H, Kempermann G, Gage FH. Neural consequences of environmental enrichment. Nat Rev Neurosci. 2000;1(3):191–8. 10.1038/35044558 11257907

[4] Rosenzweig MR, Bennett EL. Psychobiology of plasticity: effects of training and experience on brain and behavior. Behav Brain Res. 1996;78(1):57–65. 10.1016/0166-4328(95)00216-2 8793038

[5] Sale A, Berardi N, Maffei L. Enrich the environment to empower the brain. Trends Neurosci. 2009;32(4):233–9. 10.1016/j.tins.2008.12.004 19268375

[6] Coburn A, Vartanian O, Chatterjee A. Buildings, Beauty, and the Brain: A Neuroscience of Architectural Experience. J Cogn Neurosci. 2017;29(9):1521–31. 10.1162/jocn_a_01146 28493809

[7] Dzhambov AM, Markevych I, Hartig T, Tilov B, Arabadzhiev Z, Stoyanov D, et al Multiple pathways link urban green- and bluespace to mental health in young adults. Environ Res. 2018;166:223–33. 10.1016/j.envres.2018.06.004 29890427

[8] Hartig T. Green space, psychological restoration, and health inequality. Lancet. 2008;372(9650):1614–5. 10.1016/S0140-6736(08)61669-4 18994650

[9] Joye Y. Architectural lessons from environmental psychology: The case of biophilic architecture. Rev Gen Psychol. 2007;11(4):305–28.

[10] Bowler DE, Buyung-Ali LM, Knight TM, Pullin AS. A systematic review of evidence for the added benefits to health of exposure to natural environments. Bmc Public Health. 2010;10. 10.1186/1471-2458-10-456 PMC2924288 20684754

[11] Barton J, Pretty J. What is the best dose of nature and green exercise for improving mental health? A multi-study analysis. Environ Sci Technol. 2010;44(10):3947–55. 10.1021/es903183r 20337470

[12] Valtchanov D, Barton KR, Ellard C. Restorative Effects of Virtual Nature Settings. Cyberpsych Beh Soc N. 2010;13(5):503–12. 10.1089/cyber.2009.0308 20950174

[13] Berman MG, Jonides J, Kaplan S. The Cognitive Benefits of Interacting With Nature. Psychol Sci. 2008;19(12):1207–12. 10.1111/j.1467-9280.2008.02225.x 19121124

[14] Berman MG, Kross E, Krpan KM, Askren MK, Burson A, Deldin PJ, et al Interacting with nature improves cognition and affect for individuals with depression. J Affect Disorders. 2012;140(3):300–5. 10.1016/j.jad.2012.03.012 22464936 PMC3393816

[15] Berto R. Exposure to restorative environments helps restore attentional capacity. J Environ Psychol. 2005;25(3):249–59.

[16] Bratman GN, Daily GC, Levy BJ, Gross JJ. The benefits of nature experience: Improved affect and cognition. Landscape Urban Plan. 2015;138:41–50.

[17] Kaplan S. The Restorative Benefits of Nature—toward an Integrative Framework. J Environ Psychol. 1995;15(3):169–82.

[18] Kardan O, Gozdyra P, Misic B, Moola F, Palmer LJ, Paus T, et al Neighborhood greenspace and health in a large urban center. Sci Rep-Uk. 2015;5. 10.1038/srep11610 PMC4497305 26158911

[19] Pretty J, Peacock J, Hine R, Sellens M, South N, Griffin M. Green exercise in the UK countryside: Effects on health and psychological well-being, and implications for policy and planning. J Environ Plann Man. 2007;50(2):211–31.

[20] Kuo FE, Sullivan WC. Environment and crime in the inner city—Does vegetation reduce crime? Environ Behav. 2001;33(3):343–67.

[21] Kuo FE, Sullivan WC. Aggression and violence in the inner city—Effects of environment via mental fatigue. Environ Behav. 2001;33(4):543–71.

[22] Ulrich RS. View through a window may influence recovery from surgery. Science. 1984;224(4647):417–9. 10.1126/science.6200934 6143402

[23] Kellert SR, Wilson EO. The Biophilia hypothesis. Washington, D.C.: Island Press; 1993 484 p. p.

[24] Frumkin H. Beyond toxicity: human health and the natural environment. Am J Prev Med. 2001;20(3):234–40. 10.1016/s0749-3797(00)00317-2 11275453

[25] Salingaros N. Fractal Art and Architecture Reduce Physiological Stress. Journal of Biourbanism. 2012;II(2):11–28.

[26] Salingaros N. The sensory value of ornament. Communication & Cognition. 2003;36(3):331–51.

[27] Taylor RP. Reduction of Physiological Stress Using Fractal Art and Architecture. Leonardo. 2006;39(3):245–51.

[28] Goldberger AL. Fractals and the birth of Gothic: reflections on the biologic basis of creativity. Mol Psychiatry. 1996;1(2):99–104. 9118332

[29] Alexander C, Ishikawa S, Silverstein M, Jacobson M, Fiksdahl King I, Angel S. A Pattern Language. New York: Oxford University Press; 1977.

[30] Salingaros NA. The laws of architecture from a physicist’s perspective. Physics Essays. 1995;8(4):638–43.

[31] Salingaros NA. A Scientific Basis for Creating Architectural Forms. Journal of Architectural and Planning Research. 1998;15:283–93.

[32] Salingaros NA, Mehaffy MW. A theory of architecture. Solingen: Umbau-Verlag; 2006 278 pages p.

[33] Salingaros N. The Biophilic Index Predicts Healing Effects of the Built Environment. Journal of Biourbanism. 2019;8(1).

[34] Mandelbrot BB. How long is the coast of Britain? Statistical self-similarity and fractional dimension. Science. 1967;156(3775):636–8. 10.1126/science.156.3775.636 17837158

[35] Weibel ER. Fractal geometry: a design principle for living organisms. Am J Physiol. 1991;261(6 Pt 1):L361–9. 10.1152/ajplung.1991.261.6.L361 1767856

[36] Mandelbrot BB. The fractal geometry of nature. San Francisco: W.H. Freeman; 1982 460 p., 1 leaf of plates p.

[37] Bailey JK, Bangert RK, Schweitzer JA, Trotter RT 3rd, Shuster SM, Whitham TG. Fractal geometry is heritable in trees. Evolution. 2004;58(9):2100–2. 10.1554/04-151 15521465

[38] Taylor RP, Spehar B, Van Donkelaar P, Hagerhall CM. Perceptual and Physiological Responses to Jackson Pollock’s Fractals. Front Hum Neurosci. 2011;5:60 10.3389/fnhum.2011.00060 21734876 PMC3124832

[39] Joye Y. Fractal Architecture Could Be Good for You Nexus Network Journal. 2007;9(2):311–20.

[40] Salingaros N. Unified architectural theory: form, language, complexity: a companion to Christopher Alexander’s "The phenomenon of life: the nature of order, book 1". Portland, Oregon Sustasis Foundation; 2013.

[41] Coburn A, Kardan O, Kotabe H, Steinberg J, Hout MC, Robbins A, et al Psychological responses to natural patterns in architecture. J Environ Psychol. 2019;62:133–45.

[42] Berman MG, Hout MC, Kardan O, Hunter MR, Yourganov G, Henderson JM, et al The Perception of Naturalness Correlates with Low-Level Visual Features of Environmental Scenes. Plos One. 2014;9(12). 10.1371/journal.pone.0114572 PMC4273965 25531411

[43] Biederman I, Vessel EA. Perceptual pleasure and the brain. Am Sci. 2006;94(3):247–53.

[44] Stamps AE III. Mystery, complexity, legibility and coherence: A meta-analysis. J Environ Psychol. 2004;24:1–16.

[45] Kaplan R, Kaplan S. The experience of nature: A psychological perspective. Cambridge, UK: Cambrigde Unisversity Press; 1989.

[46] Kaplan R, Kaplan S, Ryan RL. With people in mind: design and management of everyday nature. Washington, D.C.: Island Press; 1998 xiv, 225 p. p.

[47] Sierpiński W. Sur une courbe cantorienne qui contient une image biunivoque et continue de toute courbe donnée. Compt Rend Acad Sci Paris. 1916(162):629–32.

[48] Sierpinski W. Sur une courbe dont tout point est un point de ramification. Compt Rend Acad Sci Paris. 1915;160:302–5.

[49] Gonzato G, Mulargia F, Ciccotti M. Measuring the fractal dimensions of ideal and actual objects: implications for application in geology and geophysics. Geophys J Int. 2000;142(1):108–16.

[50] Forsythe A, Nadal M, Sheehy N, Cela-Conde CJ, Sawey M. Predicting beauty: Fractal dimension and visual complexity in art. Brit J Psychol. 2011;102:49–70. 10.1348/000712610X498958 21241285

[51] Shemesh A, Talmon R, Karp O, Amir I, Bar M, Grobman YJ. Affective response to architecture—investigating human reaction to spaces with different geometry. Archit Sci Rev. 2017;60(2):116–25.

[52] Djebbara Z, Fich LB, Petrini L, Gramann K. Sensorimotor brain dynamics reflect architectural affordances. P Natl Acad Sci USA. 2019;116(29):14769–78. 10.1073/pnas.1900648116 PMC6642393 31189596

[53] Gobster PH, Nassauer JI, Daniel TC, Fry G. The shared landscape: what does aesthetics have to do with ecology? Landscape Ecol. 2007;22(7):959–72.

[54] Ode A, Fry G, Tveit MS, Messager P, Miller D. Indicators of perceived naturalness as drivers of landscape preference. J Environ Manage. 2009;90(1):375–83. 10.1016/j.jenvman.2007.10.013 18280633

[55] Gret-Regamey A, Bishop ID, Bebi P. Predicting the scenic beauty value of mapped landscape changes in a mountainous region through the use of GIS. Environ Plann B. 2007;34(1):50–67.

[56] Schirpke U, Timmermann F, Tappeiner U, Tasser E. Cultural ecosystem services of mountain regions: Modelling the aesthetic value. Ecol Indic. 2016;69:78–90. 10.1016/j.ecolind.2016.04.001 27482152 PMC4962904

[57] Dramstad WE, Tveit MS, Fjellstad WJ, Fry GLA. Relationships between visual landscape preferences and map-based indicators of landscape structure. Landscape Urban Plan. 2006;78(4):465–74.

[58] Jessel B. Elements, characteristics and character—Information functions of landscapes in terms of indicators. Ecol Indic. 2006;6(1):153–67.

[59] Kuper R. Evaluations of landscape preference, complexity, and coherence for designed digital landscape models. Landscape Urban Plan. 2017;157:407–21.

[60] Schirpke U, Tappeiner G, Tasser E, Tappeiner U. Using conjoint analysis to gain deeper insights into aesthetic landscape preferences. Ecol Indic. 2019;96:202–12.

[61] Hariri M. Designing perfume jewellery: A study in structured complexity. Unpublished Master’s thesis: London Metropolitan University, London, UK; 2017.

[62] Sussman A, Ward J. Eye-tracking Boston City Hall to better understand human perception and the architectural experience. New Design Ideas. 2019;3(1):53–9.

[63] Sussman A, Ward J. Planning for the Subconscious 2016 [updated June. https://www.planning.org/planning/2016/jun/subconscious/.

[64] Sussman A, Ward J. Game-Changing Eye-Tracking Studies Reveal How We Actually See Architecture 2017 [https://commonedge.org/game-changing-eye-tracking-studies-reveal-how-we-actually-see-architecture/.

[65] Simoncelli EP, Olshausen BA. Natural image statistics and neural representation. Annual Review of Neuroscience. 2001;24:1193–216. 10.1146/annurev.neuro.24.1.1193 11520932

[66] Martins MJ, Fischmeister FP, Puig-Waldmuller E, Oh J, Geissler A, Robinson S, et al Fractal image perception provides novel insights into hierarchical cognition. Neuroimage. 2014;96:300–8. 10.1016/j.neuroimage.2014.03.064 24699014

