# Supplementary figures and images for: Aesthetic preference is related to organized complexity
Source: PLoS One. 2020 Jun 26;15(6):e0235257. doi: 10.1371/journal.pone.0235257 (PMC7319303; doi:10.1371/journal.pone.0235257)

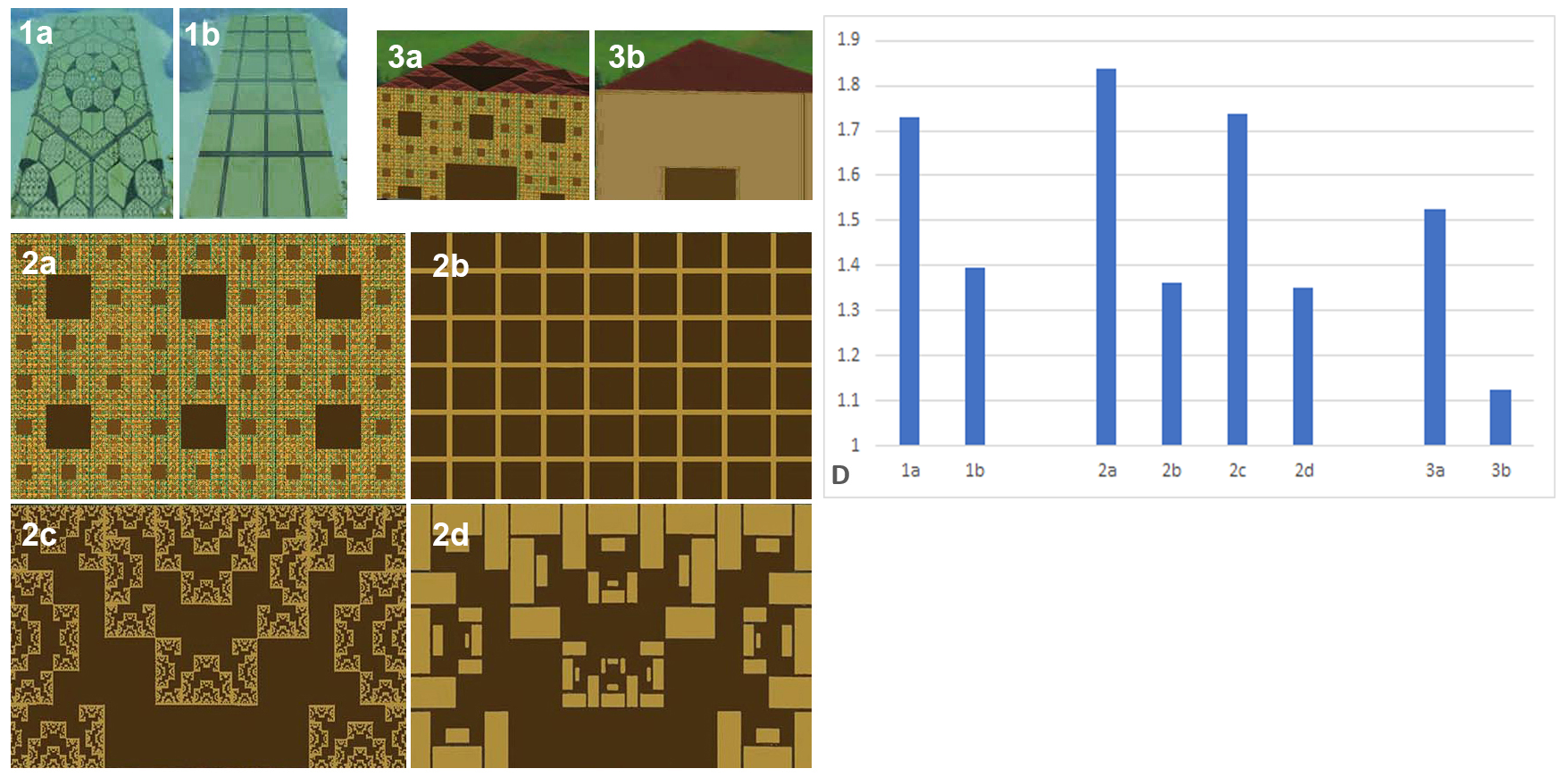

Supplement: S1 Fig — (TIF) [file pone.0235257.s001.tif]
