# Supplementary material for: Aesthetic preference is related to organized complexity
Source: PLoS One. 2020 Jun 26;15(6):e0235257. doi: 10.1371/journal.pone.0235257 (PMC7319303; doi:10.1371/journal.pone.0235257)
Supplement: S1 File — (PDF) [file pone.0235257.s002.pdf]

Table S1: Socio-demographic characteristics of the survey respondents (n=144).

**Socio-demographic characteristics**		**Number of respondents (%)**
Gender	Female	54.2
	Male	45.1
	no answer	0.7
Age	< 18 years	0.7
	18-30 years	20.1
	31-39 years	19.4
	40-49 years	26.4
	50-59 years	22.2
	60-69 years	9.0
	> 70 years	1.4
	no answer	0.7
Place of living	Large city (>200,000 residents)	58.3
	Small town (<20,000 residents)	16.0
	Town (<200,000 residents)	21.5
	Village (<1,000 residents)	4.2

Table S2: Results of the paired T-test between the individual images of each figure.

Figure	Image pairs	Paired Differences					t	df	Sig. (2-tailed)
		Mean	Std. Dev.	Std. Error Mean	95% CI				
					Lower	Upper			
1	1a - 1b	0.250	0.972	0.081	0.090	0.410	3.088	143	0.002**
2	2a - 2b	0.236	0.719	0.060	0.118	0.355	3.939	143	<0.001***
	2a - 2c	0.035	0.880	0.073	-0.110	0.180	0.473	143	0.637
	2a - 2d	0.340	0.593	0.049	0.243	0.438	6.883	143	<0.001***
	2b - 2c	-0.201	0.705	0.059	-0.318	-0.085	-3.426	143	<0.001***
	2b - 2d	0.104	0.469	0.039	0.027	0.181	2.666	143	0.009**
	2c - 2d	0.306	0.583	0.049	0.210	0.402	6.292	143	<0.001***
3	3a - 3b	0.472	0.885	0.074	0.327	0.618	6.406	143	<0.001***
4	4a - 4b	0.597	0.805	0.067	0.465	0.730	8.904	143	<0.001***
5	5a - 5b	-0.313	0.684	0.057	-0.425	-0.200	-5.481	143	<0.001***
	5a - 5c	-0.313	0.684	0.057	-0.425	-0.200	-5.481	143	<0.001***
	5b - 5c	0.000	0.939	0.078	-0.155	0.155	0.000	143	1.000
6	6a - 6b	0.486	0.748	0.062	0.363	0.609	7.800	143	<0.001***
	6a - 6c	0.528	0.689	0.057	0.414	0.641	9.192	143	<0.001***
	6a - 6d	0.542	0.668	0.056	0.432	0.652	9.735	143	<0.001***
	6b - 6c	0.042	0.514	0.043	-0.043	0.126	0.973	143	0.332
	6b - 6d	0.056	0.499	0.042	-0.027	0.138	1.337	143	0.183
	6c - 6d	0.014	0.458	0.038	-0.062	0.089	0.364	143	0.716
7	7a - 7b	0.403	0.742	0.062	0.281	0.525	6.518	143	<0.001***
	7a - 7c	0.444	0.687	0.057	0.331	0.558	7.760	143	<0.001***
	7a - 7d	0.375	0.774	0.065	0.247	0.503	5.811	143	<0.001***
	7b - 7c	0.042	0.514	0.043	-0.043	0.126	0.973	143	0.332
	7b - 7d	-0.028	0.579	0.048	-0.123	0.068	-0.576	143	0.566
	7c - 7d	-0.069	0.537	0.045	-0.158	0.019	-1.551	143	0.123

*Significance level at $p < 0.05$.
**Significance level at $p < 0.01$.
***Significance level at $p < 0.001$.

CI = Confidence Interval of the Difference
df = degrees of freedom

Table S3: Results of the paired T-test between the four options (B = "Most beautiful", I = "Most interesting", S = "Makes more sense" and F = "Feels more familiar") for each figure.

Figure	Option	Paired Differences					t	df	Sig. (2-tailed)
		Mean	Std. Dev.	Std. Error Mean	95% CI				
					Lower	Upper			
1	B - I	-0.012	0.627	0.048	-0.107	0.083	-0.245	168	0.806
	B - F	-0.089	0.680	0.052	-0.192	0.014	-1.697	168	0.092
	B - S	-0.112	0.694	0.053	-0.218	-0.007	-2.107	168	0.037*
	I - F	-0.077	0.690	0.053	-0.182	0.028	-1.449	168	0.149
	I - S	-0.101	0.704	0.054	-0.208	0.006	-1.857	168	0.065
	F - S	-0.024	0.763	0.059	-0.140	0.092	-0.403	168	0.687
2	B - I	0.144	0.681	0.054	0.037	0.250	2.671	159	0.008**
	B - F	0.031	0.772	0.061	-0.089	0.152	0.512	159	0.609
	B - S	0.075	0.740	0.059	-0.041	0.191	1.282	159	0.202
	I - F	-0.113	0.663	0.052	-0.216	-0.009	-2.145	159	0.033
	I - S	-0.069	0.636	0.050	-0.168	0.030	-1.368	159	0.173
	F - S	0.044	0.721	0.057	-0.069	0.156	0.767	159	0.444
3	B - I	0.130	0.613	0.047	0.037	0.223	2.761	168	0.006**
	B - F	-0.059	0.761	0.059	-0.175	0.056	-1.010	168	0.314
	B - S	-0.030	0.743	0.057	-0.142	0.083	-0.517	168	0.606
	I - F	-0.189	0.645	0.050	-0.287	-0.091	-3.815	168	<0.001***
	I - S	-0.160	0.630	0.048	-0.255	-0.064	-3.297	168	0.001**
	F - S	0.030	0.782	0.060	-0.089	0.148	0.492	168	0.624
4	B - I	0.313	0.662	0.052	0.210	0.415	6.032	162	<0.001***
	B - F	0.196	0.785	0.061	0.075	0.318	3.195	162	0.002**
	B - S	0.184	0.795	0.062	0.061	0.307	2.954	162	0.004**
	I - F	-0.117	0.571	0.045	-0.205	-0.028	-2.607	162	0.010**
	I - S	-0.129	0.579	0.045	-0.218	-0.039	-2.841	162	0.005**
	F - S	-0.012	0.685	0.054	-0.118	0.094	-0.229	162	0.819
5	B - I	0.255	0.641	0.050	0.156	0.353	5.104	164	<0.001***
	B - F	0.115	0.776	0.060	-0.004	0.234	1.906	164	0.058
	B - S	0.085	0.799	0.062	-0.038	0.208	1.363	164	0.175
	I - F	-0.139	0.583	0.045	-0.229	-0.050	-3.070	164	0.003**
	I - S	-0.170	0.601	0.047	-0.262	-0.077	-3.627	164	<0.001***
	F - S	-0.030	0.728	0.057	-0.142	0.082	-0.535	164	0.593
6	B - I	0.359	0.746	0.058	0.245	0.473	6.222	166	<0.001***
	B - F	0.341	0.767	0.059	0.224	0.458	5.754	166	<0.001***
	B - S	0.383	0.718	0.056	0.274	0.493	6.902	166	<0.001***
	I - F	-0.018	0.586	0.045	-0.107	0.072	-0.396	166	0.692
	I - S	0.024	0.548	0.042	-0.060	0.108	0.565	166	0.573
	F - S	0.042	0.563	0.044	-0.044	0.128	0.961	166	0.338
7	B - I	0.358	0.765	0.060	0.240	0.475	6.006	164	<0.001***
	B - F	0.394	0.722	0.056	0.283	0.505	7.013	164	<0.001***
	B - S	0.382	0.736	0.057	0.269	0.495	6.660	164	<0.001***
	I - F	0.036	0.562	0.044	-0.050	0.123	0.831	164	0.407
	I - S	0.024	0.573	0.045	-0.064	0.112	0.543	164	0.588
	F - S	-0.012	0.541	0.042	-0.095	0.071	-0.288	164	0.774

*Significance level at $p < 0.05$.
**Significance level at $p < 0.01$.
***Significance level at $p < 0.001$.

CI = Confidence Interval of the Difference
df = degrees of freedom

Table S4: Results of the paired T-test between the image pairs, distinguished by the four options (B = "Most beautiful", I = "Most interesting", S = "Makes more sense" and F = "Feels more familiar") for each figure.

Figure	Option	Image pairs	Paired Differences					t	df	Sig. (2-tailed)
			Mean	Std. Dev.	Std. Error Mean	95% CI				
						Lower	Upper			
1	B	1a - 1b	0.750	0.672	0.119	0.508	0.992	6.313	31	<0.001***
	I	1a - 1b	0.882	0.478	0.082	0.716	1.049	10.771	33	<0.001***
	F	1a - 1b	-0.021	1.011	0.147	-0.318	0.275	-0.144	46	0.886
	S	1a - 1b	-0.176	0.994	0.139	-0.456	0.103	-1.268	50	0.211
2	B	2a - 2b	0.300	0.614	0.087	0.125	0.475	3.452	49	0.001**
	B	2a - 2c	-0.140	0.948	0.134	-0.409	0.129	-1.044	49	0.301
	B	2a - 2d	0.360	0.525	0.074	0.211	0.509	4.846	49	<0.001***
	B	2b - 2c	-0.440	0.644	0.091	-0.623	-0.257	-4.831	49	<0.001***
	B	2b - 2d	0.060	0.314	0.044	-0.029	0.149	1.353	49	0.182
	B	2c - 2d	0.500	0.544	0.077	0.345	0.655	6.499	49	<0.001***
	I	2a - 2b	0.222	0.577	0.111	-0.006	0.451	2.000	26	0.056
	I	2a - 2c	-0.185	0.879	0.169	-0.533	0.162	-1.095	26	0.284
	I	2a - 2d	0.148	0.662	0.127	-0.114	0.410	1.162	26	0.256
	I	2b - 2c	-0.407	0.636	0.122	-0.659	-0.156	-3.328	26	0.003**
	I	2b - 2d	-0.074	0.474	0.091	-0.262	0.114	-0.811	26	0.425
	I	2c - 2d	0.333	0.734	0.141	0.043	0.624	2.360	26	0.026*
	F	2a - 2b	0.289	0.787	0.117	0.052	0.525	2.463	44	0.018*
	F	2a - 2c	0.244	0.830	0.124	-0.005	0.494	1.976	44	0.054
	F	2a - 2d	0.422	0.621	0.093	0.236	0.609	4.560	44	<0.001***
	F	2b - 2c	-0.044	0.673	0.100	-0.247	0.158	-0.443	44	0.660
	F	2b - 2d	0.133	0.505	0.075	-0.018	0.285	1.773	44	0.083
	F	2c - 2d	0.178	0.535	0.080	0.017	0.338	2.231	44	0.031*
	S	2a - 2b	0.184	0.834	0.135	-0.090	0.458	1.362	37	0.181
	S	2a - 2c	0.184	0.834	0.135	-0.090	0.458	1.362	37	0.181
	S	2a - 2d	0.421	0.552	0.090	0.240	0.602	4.704	37	<0.001***
	S	2b - 2c	0.000	0.735	0.119	-0.242	0.242	0.000	37	1.000
	S	2b - 2d	0.237	0.490	0.079	0.076	0.398	2.982	37	0.005**
	S	2c - 2d	0.237	0.490	0.079	0.076	0.398	2.982	37	0.005**
3	B	3a - 3b	0.818	0.582	0.088	0.641	0.995	9.331	43	<0.001***
	I	3a - 3b	no statistics							
	F	3a - 3b	0.111	1.003	0.137	-0.163	0.385	0.814	53	0.419
	S	3a - 3b	0.306	0.962	0.137	0.030	0.582	2.228	48	0.031*
4	B	4a - 4b	0.594	0.810	0.098	0.400	0.789	6.092	68	<0.001***
	I	4a - 4b	0.778	0.647	0.152	0.456	1.099	5.102	17	<0.001***
	F	4a - 4b	0.622	0.794	0.131	0.357	0.886	4.761	36	<0.001***
	S	4a - 4b	0.538	0.854	0.137	0.262	0.815	3.939	38	<0.001***
5	B	5a - 5b	-0.367	0.802	0.104	-0.574	-0.160	-3.542	59	<0.001***
	B	5a - 5c	-0.033	0.663	0.086	-0.205	0.138	-0.389	59	0.698
	B	5b - 5c	0.333	0.837	0.108	0.117	0.550	3.085	59	0.003**
	I	5a - 5b	-0.722	0.575	0.135	-1.008	-0.437	-5.333	17	<0.001***
	I	5a - 5c	-0.111	0.471	0.111	-0.346	0.123	-1.000	17	0.331
	I	5b - 5c	0.611	0.778	0.183	0.224	0.998	3.335	17	0.004**
	F	5a - 5b	-0.268	0.593	0.093	-0.455	-0.081	-2.899	40	0.006**
	F	5a - 5c	-0.512	0.637	0.100	-0.713	-0.311	-5.147	40	<0.001***
	F	5b - 5c	-0.244	0.943	0.147	-0.542	0.054	-1.656	40	0.105
	S	5a - 5b	-0.130	0.542	0.080	-0.291	0.031	-1.632	45	0.110
	S	5a - 5c	-0.609	0.649	0.096	-0.801	-0.416	-6.361	45	<0.001***
	S	5b - 5c	-0.478	0.836	0.123	-0.727	-0.230	-3.878	45	<0.001***
6	B	6a - 6b	0.598	0.754	0.081	0.437	0.758	7.392	86	<0.001***
	B	6a - 6c	0.690	0.597	0.064	0.562	0.817	10.780	86	<0.001***

Figure	Option	Image pairs	Paired Differences					t	df	Sig. (2-tailed)
			Mean	Std. Dev.	Std. Error Mean	95% CI				
						Lower	Upper			
	B	6a - 6d	0.747	0.463	0.050	0.648	0.846	15.051	86	<0.001***
	B	6b - 6c	0.092	0.473	0.051	-0.009	0.193	1.812	86	0.073
	B	6b - 6d	0.149	0.390	0.042	0.066	0.232	3.577	86	<0.001***
	B	6c - 6d	0.057	0.279	0.030	-0.002	0.117	1.919	86	0.058
	I	6a - 6b	0.481	0.643	0.124	0.227	0.736	3.893	26	<0.001***
	I	6a - 6c	0.259	0.903	0.174	-0.098	0.616	1.492	26	0.148
	I	6a - 6d	0.481	0.643	0.124	0.227	0.736	3.893	26	<0.001***
	I	6b - 6c	-0.222	0.577	0.111	-0.451	0.006	-2.000	26	0.056
	I	6b - 6d	0.000	0.392	0.075	-0.155	0.155	0.000	26	1.000
	I	6c - 6d	0.222	0.577	0.111	-0.006	0.451	2.000	26	0.056
	F	6a - 6b	0.567	0.626	0.114	0.333	0.800	4.958	29	<0.001***
	F	6a - 6c	0.600	0.563	0.103	0.390	0.810	5.835	29	<0.001***
	F	6a - 6d	0.367	0.890	0.162	0.034	0.699	2.257	29	0.032*
	F	6b - 6c	0.033	0.320	0.058	-0.086	0.153	0.571	29	0.573
	F	6b - 6d	-0.200	0.551	0.101	-0.406	0.006	-1.989	29	0.056
	F	6c - 6d	-0.233	0.504	0.092	-0.422	-0.045	-2.536	29	0.017*
	S	6a - 6b	0.217	0.850	0.177	-0.150	0.585	1.226	22	0.233
	S	6a - 6c	0.348	0.714	0.149	0.039	0.657	2.336	22	0.029*
	S	6a - 6d	0.348	0.714	0.149	0.039	0.657	2.336	22	0.029*
	S	6b - 6c	0.130	0.626	0.130	-0.140	0.401	1.000	22	0.328
	S	6b - 6d	0.130	0.626	0.130	-0.140	0.401	1.000	22	0.328
	S	6c - 6d	0.000	0.522	0.109	-0.226	0.226	0.000	22	1.000
7	B	7a - 7b	0.591	0.689	0.073	0.445	0.737	8.048	87	<0.001***
	B	7a - 7c	0.636	0.610	0.065	0.507	0.766	9.788	87	<0.001***
	B	7a - 7d	0.591	0.689	0.073	0.445	0.737	8.048	87	<0.001***
	B	7b - 7c	0.045	0.426	0.045	-0.045	0.136	1.000	87	0.320
	B	7b - 7d	0.000	0.479	0.051	-0.102	0.102	0.000	87	1.000
	B	7c - 7d	-0.045	0.426	0.045	-0.136	0.045	-1.000	87	0.320
	I	7a - 7b	0.103	0.860	0.160	-0.224	0.430	0.648	28	0.522
	I	7a - 7c	0.207	0.774	0.144	-0.087	0.501	1.440	28	0.161
	I	7a - 7d	0.345	0.614	0.114	0.111	0.578	3.025	28	0.005**
	I	7b - 7c	0.103	0.724	0.135	-0.172	0.379	0.769	28	0.448
	I	7b - 7d	0.241	0.577	0.107	0.022	0.461	2.254	28	0.032*
	I	7c - 7d	0.138	0.516	0.096	-0.058	0.334	1.440	28	0.161
	F	7a - 7b	0.304	0.635	0.132	0.030	0.579	2.299	22	0.031*
	F	7a - 7c	0.391	0.499	0.104	0.176	0.607	3.761	22	0.001**
	F	7a - 7d	-0.130	0.968	0.202	-0.549	0.288	-0.646	22	0.525
	F	7b - 7c	0.087	0.288	0.060	-0.038	0.212	1.447	22	0.162
	F	7b - 7d	-0.435	0.662	0.138	-0.721	-0.148	-3.148	22	0.005**
	F	7c - 7d	-0.522	0.511	0.106	-0.743	-0.301	-4.899	22	<0.001***
	S	7a - 7b	0.360	0.638	0.128	0.097	0.623	2.823	24	0.009**
	S	7a - 7c	0.200	0.816	0.163	-0.137	0.537	1.225	24	0.233
	S	7a - 7d	0.200	0.816	0.163	-0.137	0.537	1.225	24	0.233
	S	7b - 7c	-0.160	0.554	0.111	-0.389	0.069	-1.445	24	0.161
	S	7b - 7d	-0.160	0.554	0.111	-0.389	0.069	-1.445	24	0.161
	S	7c - 7d	0.000	0.707	0.141	-0.292	0.292	0.000	24	1.000

*Significance level at $p < 0.05$.
**Significance level at $p < 0.01$.
***Significance level at $p < 0.001$.

CI = Confidence Interval of the Difference
df = degrees of freedom
